# Procedures Used in Managing SARS-CoV-2 Infected Dental Personnel or Patients: A Case Study From a Thai Dental Hospital

**DOI:** 10.3389/froh.2021.750394

**Published:** 2021-10-25

**Authors:** Pisha Pittayapat, Ruchanee Ampornaramveth, Chongpean Jirachoksopon, Kanchana Suvarnbriksha, Siripun Kattapong, Theerabhorn Pethprasert, Kajorn Kungsadalpipob, Soranun Chantarangsu, Panida Thanyasrisung, Natthavoot Koottathape, Suphot Tamsailom, Pairoj Linsuwanont, Kasekarn Kasevayuth, Rangsima Sakoolnamarka, Thanaphum Osathanon, Pornchai Jansisyanont

**Affiliations:** Faculty of Dentistry, Chulalongkorn University, Bangkok, Thailand

**Keywords:** COVID-19, dental practice, procedure, infection, SARS-CoV-2

## Abstract

SARS-CoV-2 can transmit undetected from asymptomatic and pre-symptomatic patients in dental clinics. Triaging dental patients using temperature and questionnaire screening cannot completely exclude asymptomatic SARS-CoV-2 infected individuals. Hence, asymptomatic SARS-CoV-2 infected individuals might visit dental hospitals/clinics seeking dental treatment without knowing that they are infected and might infect others, especially in a pandemic area. Ideally, a nasopharyngeal swab for real-time polymerase chain reaction or rapid antigen screening for dental personnel and patients prior to their appointment should be done. However, the implementation of this approach is impractical in some situations. Here, we describe the procedures for dental hospitals/clinics in case of an asymptomatic SARS-CoV-2 infected individual involved in dental service/treatment and later after testing positive for SARS-CoV-2. Potential closely contacted individuals were traced and classified according to their exposure risk. The recommended course of action is to identify individuals based on their risk and take the risk-appropriate action. We also discuss the implementation of these procedures in a dental setting during the COVID-19 pandemic in our school as a case study.

## Introduction

The first SAR-CoV-2 infected case was reported in Wuhan, China in December 2019 [1]. The number of patients rapidly increased. SAR-CoV-2 infection causes a respiratory disease that can lead to pneumonia and death. This disease was later named COVID-19. The World Health Organization declared COVID-19 a pandemic in March 2020 [2]. The pandemic presented a challenge for public health, the economy, and society. In August 2021, four SAR-CoV-2 variants are of concern; Alpha (B.1.1.7), Beta (B.1.351.2, B.1.352.3), Delta (B.1.617.2, AY.1, AY.2, AY.3), and Gamma (P.1, P.1.1, P.1.2) [3]. The major routes of SAR-CoV-2 transmission are respiratory droplets and contact transmission [4]. Dental personnel are at risk because many dental treatment procedures generate aerosols and droplets that may be contaminated with the virus [4].

The first SARS-CoV-2 positive case in Thailand was reported in January 2020. The first local infection was a taxi driver who was potentially infected from Chinese tourists [5]. Since then until now (August 2021), three waves of the COVID-19 outbreak have been reported. According to the COVID-19 dashboard by the Center for Systems Science and Engineering at Johns Hopkins University [6] and information from the Department of Disease Control, Ministry of Public Health, Thailand there have been 1,174,091 confirmed cases and 11,143 deaths as of August 29, 2021 [7]. Bangkok is the city with the highest number of reported cases in Thailand.

During the COVID-19 pandemic, the Faculty of Dentistry, Chulalongkorn University adjusted its dental services based on the control guidelines of the COVID-19 pandemic in Thailand. When the number COVID-19 cases rapidly increased, the Faculty limited dental treatment to patients requiring emergency or urgent care [8]. The protocol for preventing SARS-CoV-2 spread during dental treatment was strictly applied. The standard, contact, droplet, and airborne precautions were reconsidered and emphasized, which included patient screening, wearing appropriate personal protective equipment (PPE), strictly performing surface disinfection, limiting aerosol-generating procedures, effectively implementing local source control of dental aerosols, and improving the ventilation in our dental hospital [4, 9]. The preventive measures implemented by dental care providers are employed in our dental hospital. All personnel and individuals are required to wear a mask at all times in the hospital and public areas. Social distancing must be performed during all activities. Dental care providers who perform aerosol generating procedures must wear a cloth gown covered with a water-proof isolation gown, disposable hair cover, disposable shoe covers, N-95 mask, and face shield [10].

The percentage of asymptomatic cases in individuals testing positive for SARS-CoV-2 varies among studies [11–13]. A study of 565 evacuees from Wuhan demonstrated an asymptomatic ratio of 30.8% [11]. Notably, SARS-CoV-2 infected individuals can transmit the virus to others during the pre-symptomatic or asymptomatic periods [14].

The recommended patient screening protocol of measuring body temperature and risk history taking cannot effectively identify infected individuals, especially those without symptoms [10]. Ideally, pre-screening dental patients with a SARS-CoV-2 nasopharyngeal swab for real-time polymerase chain reaction or antigen rapid test was suggested, however, the expense might be prohibitive [15]. The use of a nasopharyngeal swab for real-time polymerase chain reaction test is impractical in some situations due to the high cost, such as when patients are required to frequently visit the dental clinic to continue their treatment. As of August 30, 2021, the Faculty of Dentistry, Chulalongkorn University, Bangkok, Thailand began requiring the SARS-CoV-2 antigen rapid test for patients requiring aerosol-generating procedures. In addition, the faculty encourages a routine use the SARS-CoV-2 antigen rapid test for screening dental care providers and dental hospital personnel. However, there are a few limitations to rapid antigen testing, e.g., false negative results due to the incubation period, the sensitivity and specificity of a particular test kit, and poor sample collection technique [16, 17]. Screening with rapid antigen testing would be beneficial to exclude asymptomatic SARS-CoV-2 infected individuals from receiving treatment at that time. However, this screening procedure is not mandatory in general dental treatment in Thailand because patients are not required to undergo a nasopharyngeal swab test prior to dental treatment. Thus, it is inevitable that dental hospitals/clinics will treat pre-symptomatic or asymptomatic COVID-19 patients. Similarly, dental personnel, i.e., dentists, dental hygienists, dental assistants, and receptionists, could also be infected without developing symptoms and perform their duties without knowing that they are infected. Although a daily screening protocol for dental staff and self-monitoring for signs and symptoms of SARS-CoV2 infection can be enacted, dental personnel with an asymptomatic SARS-CoV2 infection might still report for work. Once symptoms develop and an individual tests positive for SARS-CoV-2, the appropriate protocols to identify the exposure risks of any dental staff and patients that had come in close contact with that person should be promptly applied.

Procedure guidelines should explicitly classify the risk of closely contacted persons. Here, we describe our procedural guidelines to manage the situation after receiving a report of a SARS-CoV-2 infected dental personnel and/or patients after visiting the dental hospitals/clinics. Initially, a timeline investigation of the confirmed cases in the dental hospitals/clinics is conducted. Subsequently, the closely contacted individuals are identified and categorized according to their risk of exposure. These individuals must be notified and informed of the appropriate guidelines for preliminary self-care and monitoring. The potentially contaminated area where the indexed case was at has to be evaluated and properly disinfected. Importantly, the appropriate information should be effectively disseminated in the organization and public. The implementation of this procedural guideline in the Faculty of Dentistry, Chulalongkorn University is also described as a case study. The aim of the present case study was to share our procedures to prevent and monitor the SAR-CoV-2 spread in the dental hospital and this could be further applied and modified to surveil the potential transmission in other dental clinical setting.

## Case Study

### Case Presentation

**Figure 1:**
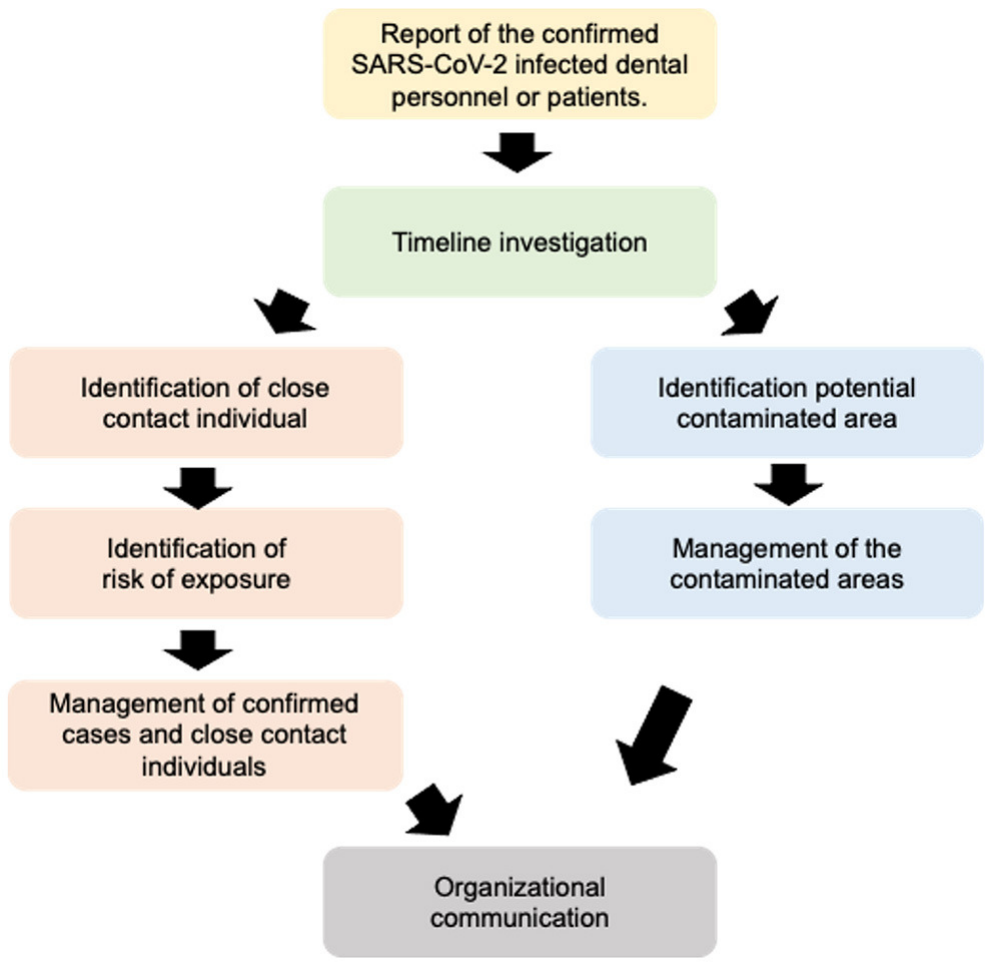
Flow chart illustrating the guideline sequence.

The Committee for COVID-19 Pandemic Monitoring, Faculty of Dentistry, Chulalongkorn University developed procedure guidelines to be implemented when a confirmed SARS-CoV-2 infected dental personnel or patient is reported. The procedure guidelines were modified from the Department of Disease Control of Thailand [18]. The management was divided into four sections (Figure 1): (1) timeline investigation, (2) management of confirmed cases and close contact individuals, (3) management of the contaminated areas, and (4) organizational communication. This guideline was applied in the Faculty of Dentistry, Chulalongkorn University, Bangkok, Thailand.

### Timeline Investigation

The timeline investigation has to be performed effectively and in a timely manner. Careful investigation is required to identify those who were in close contact with the confirmed case and the potentially contaminated area. The potential exposure period, symptomatic and/or tested positive period should be determined. The contacted persons are defined as those having common activities with the confirmed case and can be categorized into (1) the closely contacted persons with the confirmed case and (2) the contacted persons who are potentially exposed to SARS-CoV-2 from the confirmed case. The contacted persons who are a potential source of infection are investigated to determine the potential infection in the dental hospitals/clinic areas. The contacted persons who are potentially exposed to SARS-CoV-2 are the main targets for the timeline investigation to rapidly prevent further spread. This category comprises individuals in contact with the confirmed case on the date with symptoms (or 1–2 days prior to the date of developing symptoms). The closely contacted individual are defined as (1) individuals who were closer than 1 meter from the confirmed case for more than 5 min, and (2) individuals who were in a poorly ventilated area together with the confirmed case for more than 15 min. The individuals in close contact are categorized according to their exposure risk; high, moderate, or low risk. Several factors are considered as criteria e.g., properly wearing PPE, types of masks, distance, ventilation, and close contact time. The high risk category includes (a) individuals who did not properly wear PPE and have the potential to be directly exposed to respiratory secretions, coughs, or sneezes from the confirmed case, (b) individuals who were directly contacted by contaminated materials, (c) Individuals who had daily-life activities together, such as having meals together, with the confirmed cases and may not have properly worn PPE all the time, (d) patients who received dental treatment in the same room after the treatment of a confirmed case that day, despite proper ventilation and disinfection protocols, and (e) patients who received treatment from dental personnel who tested positive for SARS-CoV-2, despite proper PPE wearing and disinfection protocols. Moderate risk individuals include (a) individuals who are in close contact (<1 m) for more than 15 min with the confirmed case but wore surgical masks and (b) dental personnel who worked on the case with the dental personnel testing positive for SARS-CoV-2, despite appropriately wearing PPE. Lastly, low risk individuals include (a) individuals who were in the same area further than 1 m from the confirmed case and wore surgical masks and (b) individuals who had a brief communication with the confirmed case (<5 min) and wore surgical masks.

### Management of Confirmed Cases and Close Contact Individuals

After identifying the exposure risk of closely contacted individuals, the appropriate management must be administered. The Committee for COVID-19 Pandemic Monitoring, Faculty of Dentistry, Chulalongkorn University established the procedure guidelines for individuals with high, moderate, and low risk of exposure as indicated in Table 1. For high-risk individuals, a nasopharyngeal swab SARS-CoV-2 test will be performed. The cost of the nasopharyngeal swab SARS-CoV-2 test is supported by the Faculty and the University for all patients and personnel. If the individuals receive negative results, they must home-quarantine for 14 days to observe their symptoms. After the 14-day quarantine, the individuals can return to work if they did not develop any symptoms during the quarantine period. It is suggested that the individuals get a nasopharyngeal swab SARS-CoV-2 test prior to resuming their work in patient care. However, due to limited resources, we did not re-test all individuals after the 14-day quarantine.

**Table 1:** Procedure guidelines for individuals with high, moderate, and low risk of exposure.

**Category**	**Procedure guideline**
High risk	1. Strictly quarantine for 14 days from the date of exposure to the confirmed case. Quarantine immediately after receiving notification of their exposure. 2. Have a SARS-CoV-2 test using a nasopharyngeal swab (suggested to test 5 days after the date of exposure to the confirmed case.) 3. When the test result in (2) is negative and later during quarantine the individual develops symptoms, they must retest for SARS-CoV-2 using a nasopharyngeal swab. 4. When the test result in (2) is negative and after quarantine for 14 days without any symptoms, the individual can return to work and strictly follow the infection control guidelines.
Moderate risk	1. Strictly quarantine for 14 days from the date of exposure to the confirmed case. Quarantine immediately after receiving notification of their exposure. 2. If an individual develops any symptoms during the quarantine period they must test for SARS-CoV-2 using a nasopharyngeal swab. 3. When an individual is in quarantine for 14 days without any symptoms, they can return to work and strictly follow the infection control guidelines.
Low risk	1. Individuals can work normally and strictly follow the infection control guidelines. However, individuals should avoid crowded areas and wear surgical masks. 2. Individuals must self-monitor for any symptoms for 14 days. If they develop any symptoms during the self-monitoring period, they must test for SARS-CoV-2 using a nasopharyngeal swab.

### Management of the Contaminated Areas

Based on the timeline investigation, the potentially contaminated area can be identified. A high risk contaminated area is closed for use until the appropriate management is applied [10]. The contaminated area has to be correctly sprayed with disinfectant and disinfected with surface cleaning [10]. The area is managed to have appropriate air ventilation prior to the next use.

### Organizational Communication

The organizational communication regarding the situation has to be announced periodically [10]. This communication aims to raise the awareness of the dental personnel, while not inducing panic among the staff. Hence, the communication focuses on the route of transmission that occurred among the dental personnel to increase consciousness of these specific issues. In addition, the procedures and infection control guideline must be routinely reinforced to the dental hospital community.

### Procedure Implementation and Current Status of Our Faculty and Dental Hospital

During the first wave of the outbreak, our school received a report of a part-time lecturer who tested positive for SAR-CoV-2 in March 2020. Our investigation indicated that the individual had been infected from an unknown source outside the school. At that time, all contacted persons at the dental school were identified. These individuals tested negative for SAR-CoV-2 and none developed any symptoms during the quarantine period. During the third wave of the COVID-19 outbreak in Thailand, the Faculty of Dentistry, Chulalongkorn University Dental Hospital limited it services to only emergency and urgent dental care [8]. The procedure described above was applied in the Faculty of Dentistry, Chulalongkorn University, Bangkok, Thailand beginning in April 2021. From April 16 to June 11, 2021, 22 general practitioners and nine radiologists were rotated on duty. In addition, 74 dental specialists served on an on-call basis. Total of 358 other personnel were rotated on duty, for example nurses, dental assistants, pharmacists, cleaning staffs, and back-office personnel. Patients were required to consult with dentists via online application. After dentists made preliminary diagnosis and designed that patient were required dental treatment, patients were then received the appointment to visit the dental hospital. One thousand nine hundred and forty five patients required dental services during April 16 to June 11, 2021. Aerosol-generating procedures were performed on 19.59% of these patients. Seven infected patients receive dental treatment while asymptomatic and subsequently informed their dentist that they had tested positive for SAR-CoV-2.

During April until June 30, 2021, 23 dental personnel (3 dentists, 8 undergraduate students, 9 dental assistants, and 3 other hospital personnel) tested positive for SAR-CoV-2 via the nasopharyngeal swab test. Their signs and symptoms included cough, fever, sore throat, running nose, stuffy nose, headache, body aches, nausea and vomiting, loss of taste and smell, and diarrhea. Fourteen out of 23 confirmed cases were infected outside the dental school, e.g., from visiting a high-risk area, or entertainment venue. The Committee performed the procedure guidelines as described above. The closely contacted individuals were identified according to their risk of exposure. Three hundred and three people received a nasopharyngeal swab to determine if they were infected with SAR-CoV-2. Nine out of 303 closely contacted persons tested positive for SAR-CoV-2. The procedure guidelines were then further applied to the positive closely contacted persons for identifying potential infected individuals. In these cases, transmission among the dental personnel in the school was potentially from close contact during daily activities, such as having lunch together without proper social distancing and not wearing a mask.

Due to using the appropriate infection control protocols for preventing SAR-CoV-2 spread, we did not receive any report of potential SAR-CoV-2 transmission from a patient to dental personnel or from dental personnel to a patient from dental treatment procedures.

## Discussion

The procedure guideline described in the present case study was modified from the general guideline of the Department of Disease Control of Thailand [18]. There is no other specific guideline for dental hospitals in Thailand. Hence, the effectiveness and benefit of these current procedures cannot be described in comparison to other procedures. However, based on our dental hospital experience, we effectively controlled and monitored the transmission in our hospital. There was no identified infection that occurred during dental treatment in our hospital and there was no uncontrolled SAR-CoV-2 spread in our clinical setting. In addition, our school hosted a public seminar to present our procedural guidelines for the Thai dental community to demonstrate them as a case study that can be further implemented in their dental practice.

In the present case study, there was no report of SAR-CoV-2 transmission from dental treatment procedures. This result may be due several factors. The school limited treatment to those requiring urgent or emergency treatment when there was a high rate of confirmed cases in Thailand. Furthermore, the school installed equipment to improve the ventilation and air disinfection in the dental clinic. Finally, wearing the appropriate PPE was strictly enforced and universal infection control and disinfection were routinely performed.

Our experience is similar to that found in other Asian counties. Two case reports from Singapore described that dental personnel who inadvertently performed aerosol-generating procedures (scaling and root canal treatment) on patients who had tested positive for COVID-19 did not become infected after exposure and quarantine [19]. A study from Hangzhou, China investigated the SARS-CoV-2 detection and serological IgG/IgM testing of 757 hospital staff (dentists, nurses, students, security guards, and cleaners) at the Affiliated Stomatology Hospital, Zhejijang University School of Medicine after reopening after the lockdown period [20]. This study reported that 49,007 patients visited the hospital and some of those underwent aerosol-generating procedures. Pre- and peri-operative COVID-19 preventive procedures for the dental setting were performed according to the situation described [20]. Their results demonstrated that none of the hospital staff tested positive after RT-qPCR as well as IgG and IgM testing [20]. However, it should be noted that the sensitivity and specificity of the testing methods could have generated false negatives, which could be confounding factors in this study [20]. Furthermore, there was no COVID-19 transmission found between patients and dental personnel after dental practice in Hong Kong resumed [21]. A retrospective study in Yichang, China reported that there were no confirmed cases of COVID-19 transmission due to dental procedures in the stomatology department that provided treatment while strictly adhering to cross-infection prevention protocols [22].

In contrast to the reports describing no COVID-19 transmission due to dental treatment, the Indonesian Medical Association reported that six dentists died from COVID-19. However, there were no details on how and where these dental personnel were infected [23, 24]. Therefore, an appropriate and structured study must be performed to confirm this finding. The continuous reevaluation of the procedure guidelines should be performed to implement them appropriately in the specific circumstances.

## Conclusion

The present article described the procedures used in managing a report of SARS-CoV-2 infected dental personnel or patients at the Faculty of Dentistry, Chulalongkorn University, Bangkok, Thailand. With this developed procedure, we can identify potentially infected individuals and limit the spread in the dental treatment environment. This procedure could be further implemented in other dental clinics to identify and monitor the transmission risk among dental patients and personnel. During the COVID-19 pandemic in Thailand, the potential SAR-CoV-2 transmission from a patient to dental personnel or from dental personnel to a patient from dental treatment procedures was not reported in our school. We learned from the present study that effective and timely management for identifying potentially infected individuals is beneficial to control the spread and to monitor the contaminated person/area in the dental hospital. Aerosol-generating dental treatment procedures have a high risk of transmitting SAR-CoV-2 during dental treatment. However, strictly enforced universal infection control and disinfection can prevent transmission in the dental setting. Hence, these strict infection control procedures must be routinely performed. Improved ventilation and air disinfection in the dental clinic is also strongly recommended.

## Author Contributions

PP, RA, and TO contributed to design and draft the paper. All authors contributed to data extraction and analysis of the paper content and approved the final version for publication.

## Conflict of Interest

The authors declare that the research was conducted in the absence of any commercial or financial relationships that could be construed as a potential conflict of interest.

## Publisher's Note

All claims expressed in this article are solely those of the authors and do not necessarily represent those of their affiliated organizations, or those of the publisher, the editors and the reviewers. Any product that may be evaluated in this article, or claim that may be made by its manufacturer, is not guaranteed or endorsed by the publisher.
